# Movement Disorders in Elderly Users of Risperidone and First Generation Antipsychotic Agents: A Canadian Population-Based Study

**DOI:** 10.1371/journal.pone.0064217

**Published:** 2013-05-16

**Authors:** Irina Vasilyeva, Robert G. Biscontri, Murray W. Enns, Colleen J. Metge, Silvia Alessi-Severini

**Affiliations:** 1 Faculty of Pharmacy, University of Manitoba, Winnipeg, Manitoba, Canada; 2 Department of Accounting and Finance, Asper School of Business, University of Manitoba, Winnipeg, Manitoba, Canada; 3 Department of Psychiatry, Faculty of Medicine, University of Manitoba, Winnipeg, Manitoba, Canada; University of Montreal, Canada

## Abstract

**Background:**

Despite concerns over the potential for severe adverse events, antipsychotic medications remain the mainstay of treatment of behaviour disorders and psychosis in elderly patients. Second-generation antipsychotic agents (SGAs; e.g., risperidone, olanzapine, quetiapine) have generally shown a better safety profile compared to the first-generation agents (FGAs; e.g., haloperidol and phenothiazines), particularly in terms of a lower potential for involuntary movement disorders. Risperidone, the only SGA with an official indication for the management of inappropriate behaviour in dementia, has emerged as the antipsychotic most commonly prescribed to older patients. Most clinical trials evaluating the risk of movement disorders in elderly patients receiving antipsychotic therapy have been of limited sample size and/or of relatively short duration. A few observational studies have produced inconsistent results.

**Methods:**

A population-based retrospective cohort study of all residents of the Canadian province of Manitoba aged 65 and over, who were dispensed antipsychotic medications for the first time during the time period from April 1, 2000 to March 31, 2007, was conducted using Manitoba's Department of Health's administrative databases. Cox proportional hazards models were used to determine the risk of extrapyramidal symptoms (EPS) in new users of risperidone compared to new users of FGAs.

**Results:**

After controlling for potential confounders (demographics, comorbidity and medication use), risperidone use was associated with a lower risk of EPS compared to FGAs at 30, 60, 90 and 180 days (adjusted hazard ratios [HR] 0.38, 95% CI: 0.22–0.67; 0.45, 95% CI: 0.28–0.73; 0.50, 95% CI: 0.33–0.77; 0.65, 95% CI: 0.45–0.94, respectively). At 360 days, the strength of the association weakened with an adjusted HR of 0.75, 95% CI: 0.54–1.05.

**Conclusions:**

In a large population of elderly patients the use of risperidone was associated with a lower risk of EPS compared to FGAs.

## Introduction

Major physiological changes in the aging body such as variations in body composition, metabolic capacity, and receptor functionality deeply affect the pharmacokinetics and pharmacodynamics of drugs [1], [2]. The common presence of multiple comorbid conditions further complicates the management of the elderly patient [1]. Because of these factors elderly subjects have historically been excluded from randomized controlled trials of pharmacotherapy [3]. As a result, medications are often prescribed to older patients despite the limited information available on their safety and effectiveness in the over-65 age group.

Antipsychotic agents (AA) are no exception and RCTs conducted in the elderly have been limited to patients with diagnoses of schizophrenia and dementias [4]–[6]. Nevertheless, antipsychotic medications continue to be prescribed widely to elderly persons to control behavioural and psychotic symptoms in a variety of diagnoses [7], [8].

The adverse effects of first-generation antipsychotic agents (FGAs) (e. g., haloperidol and phenothiazines), particularly cardiovascular events and movement disorders, such as extrapyramidal symptoms (EPS) and tardive dyskinesia, have been known for decades. However, concerns also have been raised on the use of the newer second-generation antipsychotic agents (SGAs) (e.g., risperidone, olanzapine, quetiapine), which were promoted as being safer than the FGAs. In fact, a significant body of literature has reported comparisons of severe adverse events in FGA- and SGA-treated elderly persons [9]–[23] and several warnings have also been issued by health agencies (Health Canada, FDA, EMA) advising of the increased risk of cerebrovascular events and death in patients with dementia treated with antipsychotic agents [24]–[28].

Nevertheless, the practice of prescribing antipsychotics to elderly patients has continued [29]–[31] and risperidone, the only SGA with an official indication for behavioural disturbances of dementia in Canada as well as in Europe and the US, remains the antipsychotic agent most commonly prescribed to the over-65 age group [31]. The superiority of SGAs in terms of lower incidence of movement disorders or EPS such as acute dystonia, akathisia, parkinsonism and tardive dyskinesia, has been recently challenged [32]. The current study was designed to evaluate in a real world setting the incidence of movement disorders in the entire population of elderly residents of a Canadian province treated for various diagnoses with either risperidone or an FGA.

## Methods

### Ethics approval

This population-based study received ethics approval from the Health Research Ethics Board of the University of Manitoba. It was conducted in compliance with the Personal Health Information Act of Manitoba and was approved by Manitoba's Health Information Privacy Committee.

### Data source

Data for this study were obtained from the administrative health care databases of the Manitoba Population Health Research Data Repository, housed at the Manitoba Centre for Health Policy. The databases include information on the entire population of the province, which has been relatively stable at approximately 1.12 million persons during the time of the study. The use of a consistent set of identifiers allows for the integration of health histories of individuals across files and time. Nearly all contacts with the provincial health care system, including physicians' visits, hospital admissions, personal care home (PCH) residence, and pharmaceutical dispensations are recorded. All registered individuals possess a 9-digit personal health identification number (PHIN), which is scrambled to protect privacy. For this study the following databases were accessed: 1) Population Registry, 2) Hospital Abstracts, 3) Medical Services, 4) Drug Product Information Network (DPIN) prescription records, 5) PCH records, 6) Vital Statistics.

Records of physician reimbursement for medical care provided are submitted under a fee-for-service arrangement, and contain physician specialty and information on patient diagnosis at the 3-digit level of the International Classification of Diseases, Clinical Modification (ICD-9- and ICD-10-CM) classification system and physician specialty. Separation abstracts for hospital services include information on ICD-9- and ICD-10-CM diagnostic codes. Records of dispensed prescriptions (DPIN), which are submitted by retail pharmacies for reimbursement by provincial drug insurance plans or for drug utilization review purposes (regardless of insurance coverage), contain data on the date of dispensing, drug name, strength, dosage form, and quantity, and the 8-digit drug identification number (DIN).

### Study design

The study used a retrospective cohort design in which elderly residents of Manitoba, who were dispensed their first antipsychotic medication between April 1, 2000 and March 31, 2007 constituted the cohort of incident users. The date of the first dispensation of an antipsychotic prescription was considered the index date. The time frame was set to ensure that all individuals who entered the cohort had no history of antipsychotic use in the five years prior to the cohort entry (as the DPIN carries prescription information from 1995 onwards), and that all individuals could be followed for at least a year (up to March 31, 2008).

### Outcomes

The primary outcome was a composite outcome of a diagnosis for a movement disorder, EPS or parkinsonism, and/or a dispensation of antiparkinson drugs at 360 days. Secondary outcomes were the composite outcomes of a diagnosis of movement disorder, EPS or parkinsonism, and/or a dispensation of antiparkinson drugs at 30, 60, 90, and 180 days. Taking into account that drug-induced movement disorders tend to be under-diagnosed [33], a single record in the Medical Services database was considered a diagnosis of a movement disorder.

### Exclusion criteria

To ensure a complete record of patients' use of the health care system, all study subjects were required to be covered by provincial health insurance for 5 years prior to the cohort entry and during the follow-up period. As the DPIN database does not include information on medications dispensed in hospitals, patients hospitalised for longer than 25% of the year prior to the cohort entry were excluded in order to avoid a possible medication misclassification bias. Similarly, patients receiving their first antipsychotic prescription right after hospital separation were excluded if this hospitalization was longer than 30 days. As emergent EPS attributable to antipsychotic exposure was the outcome of interest, persons with prior history (5 years prior to cohort entry) of Parkinson's disease, EPS and other movement disorders and/or exposure to antiparkinson medications (i.e., dopaminergic and anticholinergic agents) as well as those with a history of brain tumours were excluded.

First-generation antipsychotics available on the Canadian market at the time of the study were included (chlorpromazine, haloperidol, flupenthixol, fluphenazine, loxapine, mesoridazine, methotrimeprazine, perphenazine, pimozide, prochlorperazine, thioridazine, trifluoperazine and zuclopenthixol). The group of FGA users was compared to the group of risperidone users.

### Follow-up

All subjects included in the study were followed until an event of interest occurred, or up to one year from the index date. Individuals were censored at the time of loss of insurance coverage, a gap in prescription refill of 30 days or longer, death or the end of study. As the DPIN database does not include information on medications administered in hospitals, persons admitted to a hospital for 30 days or longer were also censored. As well, patients who switched from a FGA to a SGA (including risperidone), or from risperidone to a FGA or another SGA were censored at the time of the switch.

### Statistical analysis

Cox proportional hazard models were used to examine the effect of risperidone use on the incidence of EPS compared to FGA use. Adjustments were made to account for potential confounders. Covariates included in the model were age, sex, PCH residence, comorbid diseases and overall comorbidity burden. Data on the number of comorbid conditions were obtained from both hospital abstracts and medical services databases. Data were accessed from the 5 years prior to the index date to build a history of comorbid conditions for each person. As an overall measure of comorbidity, the sum of Aggregated Diagnostic Groups (ADGs), as defined in the Johns Hopkins ACG ® (Adjusted Clinical Group) Case-Mix System (software version 9), was assigned to each subject [34]–[37]. The use of other medications in one year prior to the cohort entry was also controlled for. In particular, adjustments were made for the use of medications associated with development of movement disorders (i.e., reserpine, methyldopa, metoclopramide, lithium, valproate, amiodarone and tetrabenazine). Furthermore, adjustments were made for the index year to control for potential changes in prescribing patterns as observed in previous studies [31], [38].

Standardized differences were calculated to identify significant differences between FGA- and risperidone-treated groups in baseline characteristics. Standardized differences greater than 0.1 were considered to represent a significant difference between groups. Crude event rates, for each comparison, were calculated using the number of events per 100 person-years.

Analyses were performed using SAS statistical software, version 9.1.3 (SAS Institute, Cary, North Carolina). All significance testing was two-sided, with 95% confidence intervals (CIs). All analyses were conducted from the remote access site of the Manitoba Centre for Health Policy located at the Faculty of Pharmacy, University of Manitoba.

## Results

Prevalence of antipsychotic use in Manitoba was previously reported for the time period of this study [38]. The highest prevalence was observed in the elderly population (age 65 and older), reaching values of 4.3% in males and 6.0% in females [38]. These values were consistent with data reported by other jurisdictions in Canada [39], Europe [40], [41] and the US [42].

**Figure 1:**
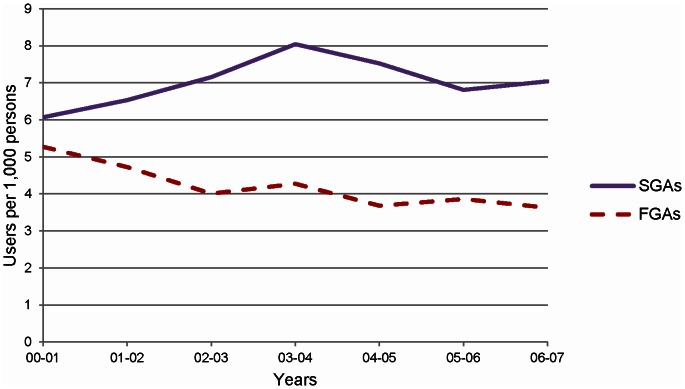
Incidence rates of antipsychotic use in the elderly population of Manitoba, 2001–2007.

**Figure 2:**
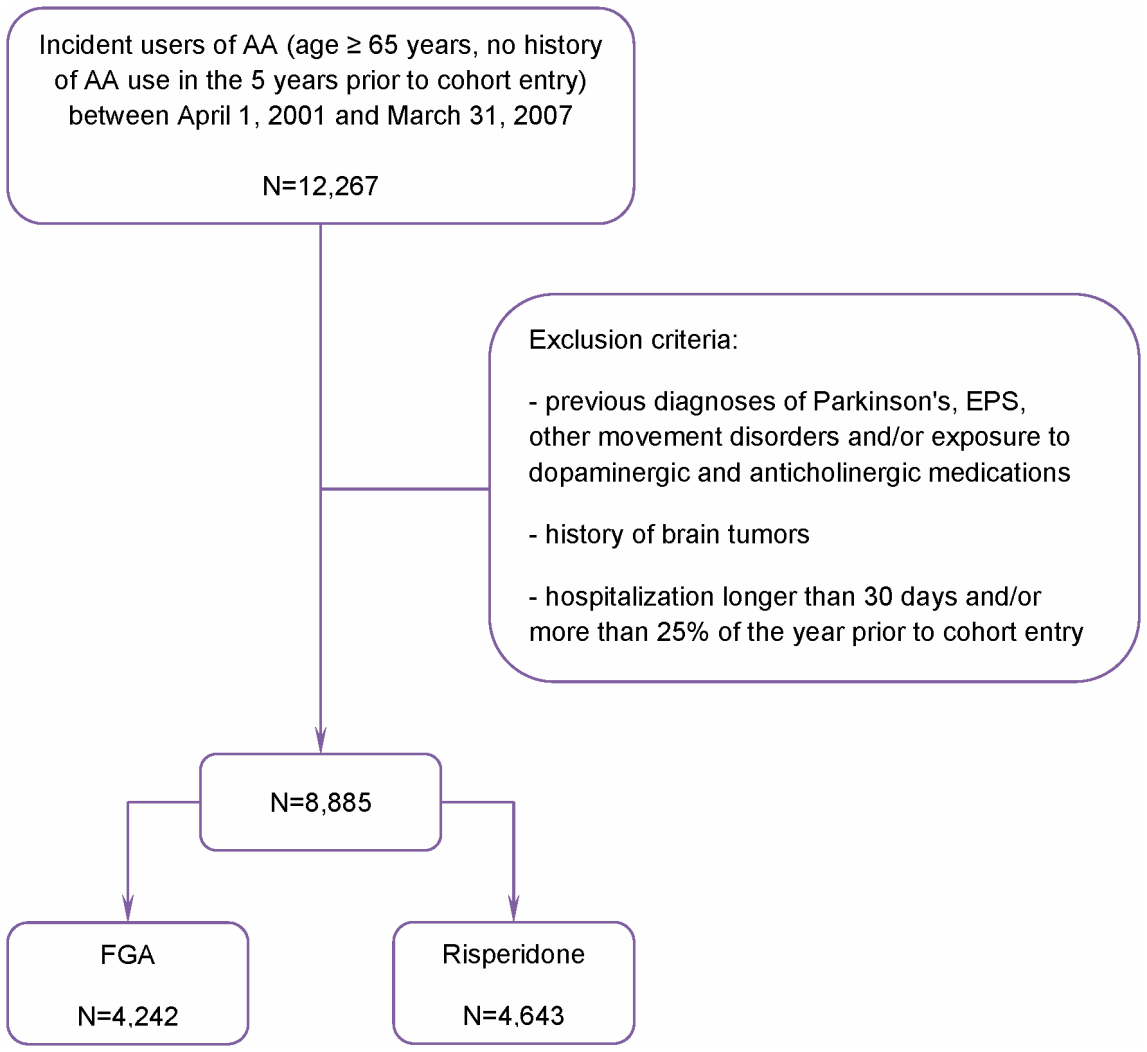
Study cohort.

Incidence rate of antipsychotic use in the elderly population of Manitoba between the fiscal year 2000–2001 and the fiscal year 2006–2007 is depicted in Figure 1. After applying all exclusion criteria a total of 8,885 persons were included in the cohort for analysis: 4,242 persons were in the FGA-exposed group and 4,643 in the risperidone-exposed group (Figure 2).

**Figure 3:**
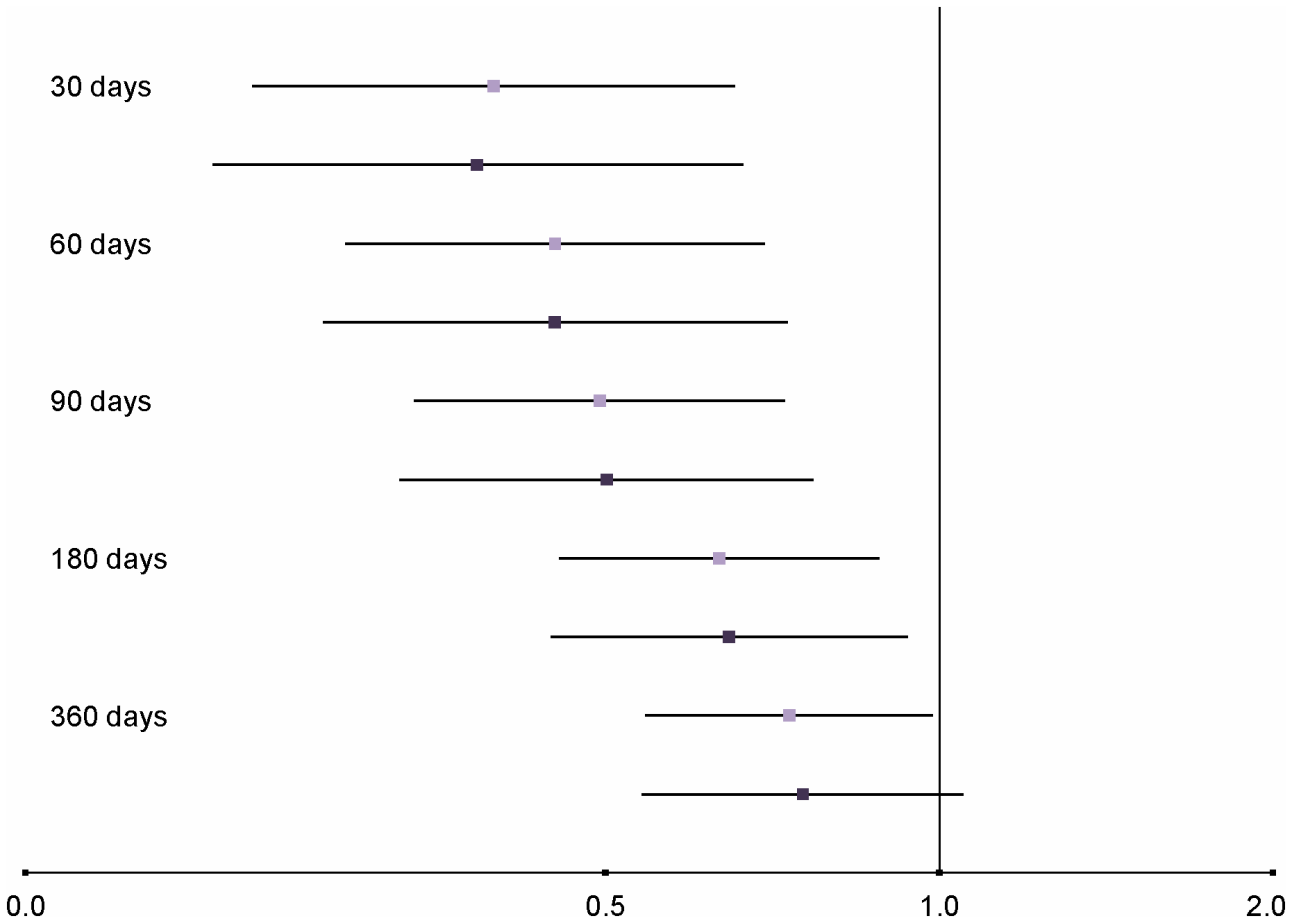
Hazard ratios for risperidone vs. FGAs. FGAs constitute the reference group. 95% CIs; unadjusted HRs in light colour square markers, adjusted in dark.

Baseline characteristics of the two groups are presented in Table 1. The number of EPS-related adverse events, mean length of follow-up and contributed person-years for FGA and risperidone users are given in Table 2. In both the unadjusted and the adjusted analyses the use of risperidone was associated with a lower risk of EPS adverse events at 30, 60, 90, and 180 days. At 360 days the adjusted HR was 0.75 with the 95% CI crossing 1.00 (0.54–1.05) (Figure 3).

**Table 1:** Baseline characteristics of FGA and risperidone users.

Characteristics	First Generation Antipsychotics N = 4,242	Risperidone N = 4,643	Standardized Difference
Age, years (mean ± SD)	77.90±7.97	83.34±7.72	0.694
Age distribution (%)			
65–74	1,682 (39.65)	710 (15.29)	
75–84	1,730 (40,78)	1,900 (40.92)	
>85	830 (19.57)	2,033 (43.79)	
Sex, male	1,816 (42.81)	1,638 (35.28)	0.155
PCH residence	637 (15.02)	1,866 (40.19)	0.587
Year of entry to cohort			
2000–2001	764 (18.01)	663 (14.28)	
2001–2002	676 (15.94)	621 (13.37)	
2002–2003	573 (13.51)	618 (13.31)	
2003–2004	623 (14.69)	680 (14.65)	
2004–2005	540 (12.73)	692 (14.90)	
2005–2006	544 (12.82)	676 (14.56)	
2006–2007	522 (12.31)	693 (14.93)	
Hospitalization in past year	2,560 (60.35)	2,222 (47.86)	0.253
Frequency of GP visits (mean ± SD)	16.02±12.74	16.42±12.89	0.031
*History of Comorbidity (%)*			
Dementia	361 (8.51)	1,666 (35.88)	0.698
Alzheimer's Disease	203 (4.79)	956 (20.59)	0.489
Schizophrenia	22 (0.52)	52 (1.12)	0.067
Delirium	104 (2.45)	301 (6.48)	0.196
Mood Disorder	118 (2.78)	286 (6.16)	0.164
Other Psychiatric Disorder	320 (7.54)	1,120 (24.12)	0.466
Stroke	100 (2.36)	202 (4.35)	0.111
*History of Medication Use*			
Number of medications used (mean ± SD)	12.48±7.60	11.31±6.94	0.161
Anticonvulsants (%)	371 (8.75)	427 (9.20)	0.016
Benzodiazepines	1,811 (42.69)	2,059 (44.35)	0.033
Antidepressants	1,073 (25.29)	1,739 (37.45)	0.264
Sedatives & Hypnotics	794 (18.72)	760 (16.37)	0.062
Anxiolytics	1,463 (34.49)	1,667 (35.90)	0.030
Acetylcholinesterase inhibitors	19 (0.45)	139 (2.99)	0.197
Other medications associated with development of movement disorders	798 (18.81)	264 (5.69)	0.409

**Table 2:** Incidence of EPS within 360 days since treatment initiation.

Cohort	No. of events	Mean duration of follow-up, days ± SD	Contributed person-years	Crude event rate, per 100 p-y
FGAs	74	88±123	1,016.79	7.27
Risperidone	111	195±140	2,472.50	4.49

## Discussion

This study provides data on the incidence of EPS adverse events in the entire elderly population of the Canadian province of Manitoba treated with antipsychotic pharmacotherapy. More than 70% of the population initiated on a SGA was prescribed risperidone. Our results show that the use of FGAs is associated with an increased risk of EPS compared to treatment with risperidone. These results are supported by biological plausibility and are consistent with the findings of previous observational studies [43]–[45]. Blockade of D_2_ receptors in the brain plays the major role in the mechanism of antipsychotic action; however, it is also associated with occurrence of EPS (specifically because of occupancy of nigrostriatal D_2_ receptors) [46]. The affinity of FGAs for dopamine D_2_ receptors leads to EPS. These agents bind more tightly than dopamine itself to the D_2_ receptors while SGAs bind less avidly than dopamine to D_2_ receptors (rapid dissociation theory) and allow normal dopamine transmission [47], [48]. The reduced blockade of D_2_ receptors has also been linked to antagonism of 5-HT_2A_ serotonin receptors. Serotonin regulates dopamine release and the presence of serotonin in nigrostriatal dopamine pathway inhibits the release of dopamine, subsequently reversing some of D_2_ blockade by SGAs [49]. Antipsychotic agents within the SGA class vary in their affinity to D_2_ receptors and risperidone has the highest affinity [47], [49].

Nevertheless, risperidone has been widely prescribed, particularly to elderly patients, with the expectation of a lower incidence of EPS compared to FGA treatment. In this study such lower risk of movement disorders has been confirmed up to approximately a year of therapy; however, a trend toward a weaker association was observed and the 95%CI of the adjusted HR at 360 days suggested a loss of statistical significance.

The interpretation of these results needs to consider the limitations of observational studies based on administrative data. However, a number of steps were taken to account for potential confounding. The key covariates that are associated with outcome or exposure were identified and adjustments were made in the analysis. Age, sex, residence in a PCH and comorbidities were used to control for a possible selection bias. The index year was included into the adjusted models to account for possible changes in medical practice over the time frame of this study. The time frame of the study cohort did not include the time period of 1998 to 2000 when much of the shift between FGA and SGA use happened.

No attempt was made to evaluate the effect of dose on incidence of EPS in this population, as small dose adjustments that commonly and continuously occur in clinical practise are not accurately captured by an observational study design that utilizes exclusively administrative data. Nevertheless, it is well known that EPS are dose-related and high doses of risperidone are expected to cause movement effects similar to those caused by FGAs.

Furthermore, this study did not address the benefits of using antipsychotic pharmacotherapy, especially because quality of life cannot be assessed in observational studies that are based on administrative data. Yet the findings of this research provide a real-world observation that the use of risperidone is associated with lower risk for potentially debilitating EPS adverse events in elderly subjects compared to the traditional antipsychotic medications. The advantage of this study is the fact that the entire elderly population of a Canadian province was included without restrictions due to insurance coverage or limited access. The results can be generalizable to other populations as they are not affected by sampling errors or recall bias. In conclusion, the information can be useful to clinicians, decision makers, patients and caregivers in choosing the most effective treatment for psychotic symptoms particularly in patients who might be at greater risk for certain adverse events such as movement disorders.

Nevertheless, the benefits of antipsychotic pharmacotherapy should always be evaluated by assessing changes in the quality of life and wellbeing of individual patients. The use and efficacy of non-pharmacological interventions remain to be evaluated in prospective studies.

## References

[1] Sitar DS (2008) Principles of medical pharmacology. In: Kalant H, Grant DM, Mitchell J, editors. Geriatric Clinical Pharmacology (7th Edition). Elsevier, ON, Canada,832840.

[2] Milton JC, Hill-Smith I, Jackson SHD (2008) Prescribing to older people. BMJ 336: 606–609. 18340075 10.1136/bmj.39503.424653.80 PMC2267940

[3] Zulman DM, Sussman JB, Chen X, Cigolle CT, Blaum CS, et al (2011) Examining the evidence: a systematic review of the inclusion and analysis of older adults in randomized controlled trials. J Gen Intern Med 26(7): 783–790. 21286840 10.1007/s11606-010-1629-x PMC3138606

[4] Ballard C, Waite J, Birks J (2006) The effectiveness of atypical antipsychotics for the treatment of aggression and psychosis in Alzheimer's disease. Cochrane Databas Syst Rev 2006 Jan 25 (1): CD003476. 10.1002/14651858.CD003476.pub2 PMC11365591 16437455

[5] McDonagh MS, Peterson K, Carson S, Fu R, Thakurta S (2010) Drug class review–Atypical Antipsychotic Drugs - Final update report–July 2010 - Oregon Health & Science University. Available: http://www.ohsu.edu/xd/research/index.cfm Accessed November 16, 2012. 21348048

[6] Ballard C, Creese B, Corbett A, Aarsland D (2012) Atypical antipsychotics for the treatment of behavioural and psychological symptoms in dementia, with a particular focus on longer term outcomes and mortality. Expert Opin Drug Saf 10(1): 35–43. 10.1517/14740338.2010.506711 20684745

[7] Chan W, Lam LC, Chen EY (2011) Recent advances in pharmacological treatment of psychosis in late life. Curr Opin Psychiatry 24: 455–460. 21799414 10.1097/YCO.0b013e32834a3f47

[8] Leslie DL, Rosenheck R (2012) Off-label use of antipsychotic medications in Medicaid. Am J Manag Care 18(3): e109–e117. 22435962

[9] Sacchetti E, Turrina C, Valsecchi P (2010) Cerebrovascular accidents in elderly people treated with antipsychotic drugs: a systematic review. Drug Saf 33(4): 273–288. 20297860 10.2165/11319120-000000000-00000

[10] Wang PS, Schneeweiss S, Avorn J, Fisher M, Mogun H, et al (2005) Risk of death in elderly users of conventional vs. atypical antipsychotic medications. N Engl J Med 353(22): 2335–2341. 16319382 10.1056/NEJMoa052827

[11] Gill SS, Bronskill SE, Normand SL, Anderson GM, Sykora K, et al (2007) Antipsychotic drug use and mortality in older adults with dementia. Ann Intern Med 146(11): 775–786. 17548409 10.7326/0003-4819-146-11-200706050-00006

[12] Schneeweiss S, Setoguchi S, Brookhart A, Dormuth C, Wang PS (2007) Risk of death associated with the use of conventional versus atypical antipsychotic drugs among elderly patients CMAJ. 176(5): 627–632. 10.1503/cmaj.061250 PMC1800321 17325327

[13] Setoguchi S, Wang PS, Brookhart AM, Canning CF, Kaci L, et al (2008) Potential causes of higher mortality in elderly users of conventional and atypical antipsychotic medications. J Am Geriatr Soc 56(9): 1644–1650. 18691283 10.1111/j.1532-5415.2008.01839.x

[14] Liperoti R, Onder G, Landi F, Lapane KL, Mor V, et al (2009) All-cause mortality associated with atypical and conventional antipsychotics among nursing home residents with dementia: a retrospective cohort study. J Clin Psychiatry 70(10): 1340–1347. 19906339 10.4088/JCP.08m04597yel PMC3775351

[15] Kales HC, Valenstein M, Kim HM, McCarthy JF, Ganoczy D, et al (2007) Mortality risk in patients with dementia treated with antipsychotics versus other psychiatric medications. Am J Psychiatry 164(10): 1568–1576. 17898349 10.1176/appi.ajp.2007.06101710

[16] Trifiro G, Verhamme KM, Ziere G, Caputi AP, Stricker BH, et al (2007) All-cause mortality associated with atypical and typical antipsychotics in demented outpatients. Pharmacoepidemiol Drug Saf 6(5): 538–544. 10.1002/pds.1334 17036366

[17] Barnett MJ, Perry PJ, Alexander B, Kaboli PJ (2006) Risk of mortality associated with antipsychotic and other neuropsychiatric drugs in pneumonia patients. J Clin Psychopharmacol 26(2): 182–187. 16633149 10.1097/01.jcp.0000203598.43314.34

[18] Rochon PA, Normand SL, Gomes T, Gill SS, Anderson GM, et al (2008) Antipsychotic therapy and short-term serious events in older adults with dementia. Arch Intern Med 168(10): 1090–1096. 18504337 10.1001/archinte.168.10.1090

[19] Ray WA, Chung CP, Murray KT, Hall K, Stein CM (2009) Atypical antipsychotic drugs and the risk of sudden cardiac death. N Engl J Med 360(3): 225–235. 19144938 10.1056/NEJMoa0806994 PMC2713724

[20] Kleijer BC, van Marum RJ, Egberts AC, Jansen PA, Knol W, et al (2009) Risk of cerebrovascular events in elderly users of antipsychotics. J Psychopharmacol 23(8): 909–914. 18635700 10.1177/0269881108093583

[21] Mehta S, Johnson ML, Chen H, Aparasu RR (2010) Risk of cerebrovascular adverse events in older adults using antipsychotic agents: a propensity-matched retrospective cohort study. J Clin Psychiatr 71(6): 689–698. 10.4088/JCP.09m05817yel 20573328

[22] Gurevich A, Guller V, Berner YN, Tal S (2012) Are typical antipsychotics safer than typical antipsychotics for treating behavioural and psychological symptoms of dementia? J Nutr Health and Aging 16(6): 557–5561. 22659997 10.1007/s12603-012-0057-5 PMC12878172

[23] Pratt N, Roughead EE, Salter A, Paul R (2012) Choice of observational study design impacts on measurement of antipsychotic risks in the elderly: a systematic review. BMC Medical Research Methodology 12: 72. Available: http://www.biomedcentral.com/1471-2288/12/72.Accessed November 16, 2012. 10.1186/1471-2288-12-72 PMC3447663 22682666

[24] Health Canada. Important drug safety information: RISPERDAL* (risperidone) and cerebrovascular adverse events in placebo-controlled dementia trials - Janssen-Ortho inc. Available: http://www.hc-sc.gc.ca/dhp-mps/medeff/advisories-avis/prof/_2002/risperdal_hpc-cps-eng.php. Accessed November 16, 2012.

[25] Health Canada. ZYPREXA* (olanzapine) and cerebrovascular adverse events in placebo-controlled elderly dementia trials. Available: http://www.hc-sc.gc.ca/dhp-mps/medeff/advisories-avis/prof/_2004/zyprexa_hpc-cps-eng.php. Accessed November 16, 2012.

[26] Health Canada. Increased mortality associated with the use of atypical antipsychotic drugs in elderly patients with dementia. Available: http://www.hc-sc.gc.ca/dhp-mps/medeff/advisories-avis/prof/_2005/atyp-antipsycho_hpc-cps-eng.php. Accessed November 16, 2012.

[27] The Food and Drug Administration (2008) FDA requests boxed warnings on older class of antipsychotic drugs. Available: http://www.fda.gov/NewsEvents/Newsroom/PressAnnouncements/2008/ucm116912.htm. Accessed November 16, 2012

[28] Drug safety update. Antipsychotics: use in elderly people with dementia. Available: http://www.mhra.gov.uk/Safetyinformation/DrugSafetyUpdate/CON088116. Accessed January 4, 2013.

[29] Valiyeva E, Herrmann N, Rochon PA, Gill SS, Anderson GM (2008) Effect of regulatory warnings on antipsychotic prescription rates among elderly patients with dementia: a population-based time-series analysis. CMAJ 179(5): 438–446. 18725616 10.1503/cmaj.071540 PMC2518182

[30] Verdoux H, Tournier M, Begaud B (2010) Antipsychotic prescribing trends: a review of pharmacoepidemiological studies. Acta Psychiatr Scand 121: 4–10. 20059452 10.1111/j.1600-0447.2009.01425.x

[31] Vasilyeva I, Biscontri RG, Enns MW, Metge CJ, Alessi-Severini S (2013) Adverse Events in Elderly Users of Antipsychotic Pharmacotherapy in the Province of Manitoba: a Retrospective Cohort Study. J Clin Psychopharmacol 33(1): 24–30. 23277238 10.1097/JCP.0b013e31827934a4

[32] Peluso MJ, Lewis W, Barnes TRE, Jones PB (2012) Extrapyramidal motor side-effects of first- and second-generation antipsychotic drugs. BJP 200: 387–392. 10.1192/bjp.bp.111.101485 22442101

[33] Esper CD, Factor SA (2008) Failure of recognition of drug-induced parkinsonism in the elderly. Movement Disorders 23(3): 401–404. 18067180 10.1002/mds.21854

[34] Starfield B, Weiner J, Mumford L, Steinwachs D (1991) Ambulatory care groups: a categorization of diagnoses for research and management. Health Serv Res 26(1): 53–74. 1901841 PMC1069810

[35] Weiner JP, Starfield BH, Steinwachs DM, Mumford LM (1991) Development and application of a population-oriented measure of ambulatory care case-mix. Med Care 29(5): 452–472. 1902278 10.1097/00005650-199105000-00006

[36] Reid RJ, MacWilliam L, Roos N, Bogdanovic B, Black B (1999) Measuring morbidity in populations: performance of the Johns Hopkins adjusted clinical group (ACG) case-mix adjustment system in Manitoba. Manitoba Centre for Health Policy. Available: http://mchp-appserv.cpe.umanitoba.ca/reference/acg.pdf Accessed November 16, 2012.

[37] The Manitoba Centre for Health Policy. Concept: Adjusted clinical group (ACG) - overview. Available: http://umanitoba.ca/faculties/medicine/units/community_health_sciences/departmental_units/mchp/resources/concept_dictionary.html. Accessed January 4, 2013.

[38] Alessi-Severini S, Biscontri RG, Collins DM, Kozyrskyj A, Sareen J, et al (2008) Utilization and costs of antipsychotic agents: a Canadian population-based study: 1996–2006. Psychiatr Serv 59 (5): 547–553. 10.1176/ps.2008.59.5.547 18451015

[39] The Canadian Institute for Health Information (2009) Antipsychotic use in seniors: an analysis focusing on drug claims, 2001 to 2007. Available: https://secure.cihi.ca/estore/productFamily.htm?pf=PFC1350&lang=en&media=0.Accessed November 16, 2012.

[40] Martinez C, Jones RW, Rietbrock S (2013) Trends in the prevalence of antipsychotic drug use among patients with Alzheimer's disease and other dementias including those treated with antidementia drugs in the community in the UK: a cohort study. BMJ Open 3:e002080. Available: http://bmjopen.bmj.com/content/3/1/e002080.Accessed January 10, 2013. 10.1136/bmjopen-2012-002080 PMC3549220 23299113

[41] Schulze J, van den Bussche H, Glaeske G, Kaduszkiewicz H, Wiese B, et al.. (2013) Impact of safety warnings on antipsychotic prescriptions in dementia: nothing has changed but the years and the substances. Eur Neuropsychopharmacol Mar 13 doi: 10.1016/j.euroneuro.2013.02.001. 10.1016/j.euroneuro.2013.02.001 23498307

[42] Kales HC, Zivin K, Kim HM, Valenstein M, Chiang C, et al (2011) Trends in antipsychotic use in dementia 1999–2007. Arch Gen Psychiatry 68(2): 190–197. 21300946 10.1001/archgenpsychiatry.2010.200

[43] Avorn J, Bohn RL, Mogun H, Gurwitz JH, Monane M, et al (1995) Neuroleptic drug exposure and treatment of parkinsonism in the elderly: a case-control study. Am J Med 99(1): 48–54. 7598142 10.1016/s0002-9343(99)80104-1

[44] Rochon PA, Stukel TA, Sykora K, Gill S, Garfinkel S, et al (2005) Atypical antipsychotics and parkinsonism. Arch Intern Med 165(16): 1882–1888. 16157833 10.1001/archinte.165.16.1882

[45] Lee PE, Sykora K, Gill SS, Mamdani M, Marras C, et al (2005) Antipsychotic medications and drug-induced movement disorders other than parkinsonism: A population-based cohort study in older adults. J Am Geriatr Soc 53(8): 1374–1379. 16078964 10.1111/j.1532-5415.2005.53418.x

[46] Thanvi B, Treadwell S (2009) Drug induced parkinsonism: a common cause of parkinsonism in older people. Postgrad Med J 85(1004): 322–326. 19528308 10.1136/pgmj.2008.073312

[47] Seeman P (2002) Atypical antipsychotics: Mechanism of action. Can J Psychiatry 47(1): 27–38. 11873706

[48] Stahl SM (2003) Describing an atypical antipsychotic: Receptor binding and its role in pathophysiology. Primary care companion J Clin Psychiatry (suppl 3): 9–13.

[49] He H, Richardson JS (1995) A pharmacological, pharmacokinetic and clinical overview of risperidone, a new antipsychotic that blocks serotonin 5-HT2 and dopamine D2 receptors. Int Clin Psychopharmacol 10(1): 19–30. 10.1097/00004850-199503000-00003 7542676

